# Evaluating the Impact of Obsessive-Compulsive Symptoms and Personality Types on Perinatal Depressive Symptoms

**DOI:** 10.3390/bs14070589

**Published:** 2024-07-11

**Authors:** Oana Neda-Stepan, Cătălina Giurgi-Oncu, Andreea Sălcudean, Elena Bernad, Brenda-Cristiana Bernad, Estera Boeriu, Virgil Radu Enătescu

**Affiliations:** 1 Doctoral School, “Victor Babes” University of Medicine and Pharmacy, Eftimie Murgu Square 2, 300041 Timisoara, Romania; oana.neda-stepan@umft.ro (O.N.-S.); bernad.brenda@umft.ro (B.-C.B.); 2 Department VIII—Neurosciences, Discipline of Psychiatry, “Victor Babes” University of Medicine and Pharmacy, Eftimie Murgu Square 2, 300041 Timisoara, Romania; catalina.giurgi@umft.ro (C.G.-O.); enatescu.virgil@umft.ro (V.R.E.); 3 Discipline of Sociobiology, Department of Ethics and Social Sciences, George Emil Palade University of Medicine, Pharmacy, Science and Technology of Targu Mures, 540136 Targu Mures, Romania; andreea.salcudean@umfst.ro; 4 Department of Obstetrics and Gynecology, “Victor Babes” University of Medicine and Pharmacy, Eftimie Murgu Square 2, 300041 Timisoara, Romania; bernad.elena@umft.ro; 5 Department of Pediatrics, “Victor Babes” University of Medicine and Pharmacy, Eftimie Murgu Square 2, 300041 Timisoara, Romania

**Keywords:** perinatal depression, personality inventory, obsessive-compulsive disorder, postpartum depression, cross-sectional studies, symptom assessment

## Abstract

Perinatal depression (PPD) presents a significant public health concern, often influenced by psychological and personality factors. This study investigated the impact of personality traits, particularly neuroticism, and obsessive-compulsive disorder (OCD) symptoms on the severity of PPD. The primary aim was to quantify the contributions of these factors to the risk and severity of PPD to enhance early intervention strategies. A total of 47 pregnant women with depressive symptoms per DSM-5 criteria at “Pius Brinzeu” County Emergency Hospital in Timisoara, Romania, were enrolled in this cross-sectional study, as well as 49 women without depressive symptoms as controls. Personality traits were assessed using the NEO Five-Factor Inventory (NEO-FFI), and OCD symptoms were measured using the Obsessive-Compulsive Inventory (OCI). Depression severity was evaluated using the Edinburgh Postnatal Depression Scale (EPDS). This set of questionnaires were administered antepartum and postpartum. The logistic regression analysis highlighted neuroticism as a significant predictor of PPD severity, with an increase in neuroticism associated with a higher risk of PPD (coefficient = 0.24, *p* < 0.001). Conversely, openness showed a protective effect (coefficient = −0.13, *p* = 0.009). Higher OCD symptomatology, particularly ordering and hoarding, were linked with increased depression scores. Specifically, the total OCI score significantly predicted the EPDS score (coefficient = 0.03, *p* = 0.003). Furthermore, significant increases in EPDS anxiety and depression scores were observed in the perinatal period, indicating worsening of symptoms (anxiety coefficient = 0.51; *p* < 0.001). The findings suggest that personality traits like neuroticism and OCD symptoms significantly contribute to the severity of PPD. Interventions targeting these specific traits could potentially mitigate the risk and severity of perinatal depression, underscoring the need for personalized treatment plans that consider these psychological dimensions.

## 1. Introduction

Perinatal depression (PPD) is a complex psychological condition affecting mothers after childbirth, characterized by significant emotional, behavioral, and physiological changes that can impact maternal health and child development [1,2]. The prevalence and impact of PPD underscore the need for a nuanced understanding of its etiological factors, which appear to be multifactorial and include biological, psychological, and social dimensions [3,4].

Personality has been widely studied as a predisposing factor in the development of various psychiatric conditions, including depression [5,6,7]. Certain personality traits, such as neuroticism, have shown strong correlations with the severity and onset of depressive symptoms [8,9,10]. In the context of perinatal depression, understanding personality types can provide insights into vulnerability and resilience factors influencing maternal mental health during the perinatal period.

Obsessive-compulsive disorder (OCD) symptoms commonly co-occur with depressive disorders and may be particularly relevant in the perinatal period due to heightened concerns about infant care and maternal competence [11,12]. The presence of OCD symptoms has been associated with increased psychological distress and may exacerbate or complicate the clinical presentation of PPD [12]. Despite considerable research on PPD, few studies have simultaneously examined the roles of personality traits and OCD symptoms in this context [13]. Nevertheless, PPD should be carefully identified and diagnosed by clinicians due to the high risk of its association with the bipolar spectrum [14].

This study hypothesizes that specific personality types, particularly those characterized by higher levels of neuroticism, and the presence of OCD symptoms are significant predictors of the severity of perinatal depression. The primary objective is to quantify the contributions of personality traits and OCD symptoms to the risk of developing perinatal depressive symptoms and PPD, aiming to identify potential targets for early intervention and support for women in the perinatal period. Through this approach, the study seeks to refine the understanding of PPD’s multifactorial etiology and enhance the specificity of predictive models in maternal mental health.

## 2. Material and Methods

### 2.1. Patients and Study Design

Sample size was calculated based on the reported prevalence of perinatal depression in the general population of approximately 10% [15,16], with a 99% confidence level, and a 10% margin of error, resulting in a minimum of 60 participants to reach significance. A total number of 96 women who were consulted in the Psychiatric Ward of “Pius Brinzeu” County Emergency hospital from Timisoara, Romania, were included in the study. From this number, 47 were pregnant women with depressive symptoms, according to DSM-5 [17], while another group of 49 women were considered the control sample. The ethical approval was received from the Ethical Board Committee of the “Pius Brinzeu” County Emergency hospital from Timisoara, Romania, affiliated with the “Victor Babes” University of Medicine and Pharmacy Timisoara (approval number 299 from 11 May 2022).

The current study followed a PICO framework to structure its investigation into perinatal depressive symptoms. P (Population) referred to women within the first year after childbirth who were recruited for this cross-sectional analysis. I (Intervention), though not a traditional intervention in the clinical sense, involved the assessment of these women using two diagnostic tools: a standardized personality inventory to evaluate personality types and the Obsessive-Compulsive Inventory (OCI) to assess the severity of OCD symptoms. C (Comparison) was made between cases and age-matched controls by mean age. O (Outcome) of the study was defined as the severity of perinatal depressive symptoms, measured by the Edinburgh Postnatal Depression Scale (EPDS). This approach enabled the researchers to systematically examine the interplay between personality traits, OCD symptoms, and their combined impact on perinatal depression severity.

### 2.2. Inclusion and Exclusion Criteria

The inclusion criteria for the current study comprised the following: (1) women within the first 6 weeks after childbirth, as the study focused on perinatal depressive symptoms; (2) women aged 18 years and older, to ensure the capability to provide informed consent and to represent a typical range for childbearing; (3) participants must have been diagnosed with perinatal depression according to DSM-5 criteria, ensuring that the study addressed its target population effectively; (4) capability of providing informed consent, confirming their willingness and understanding to participate in the study.

Exclusion criteria comprised the following: (1) women with psychiatric disorders, as these might confound the results related to perinatal depression; (2) active substance abuse problems that could interfere with the assessment or outcomes of perinatal depression; (3) chronic comorbid conditions due to their potential confounding effect on the mental health of the participants; (4) women with significant medical complications related to childbirth that could independently affect mental health status, such as severe postpartum hemorrhage or eclampsia; (5) we did not include mothers that were receiving psychological care, also due to the potential confounding effect. After the study was over, all mothers received subsequent psychiatric referral and treatment where necessary.

### 2.3. Surveys

The surveys were administered approximately two weeks before birth (antepartum) and up to six weeks postpartum. To evaluate the personality profiles of participants, the NEO Five-Factor Inventory (NEO-FFI) was utilized [18]. This inventory is a concise measure of the five major domains of personality: Neuroticism, Extraversion, Openness to Experience, Agreeableness, and Conscientiousness. The NEO-FFI consists of 60 items, each rated on a Likert scale from 0 (strongly disagree) to 4 (strongly agree). The NEO-FFI is widely recognized for its psychometric robustness and has been validated across diverse populations, making it an appropriate choice for assessing stable personality traits that may influence vulnerability to PPD.

The severity of OCD symptoms was assessed using the Obsessive-Compulsive Inventory (OCI) [19]. This self-report scale comprises 42 items that describe common obsessive-compulsive symptoms across multiple dimensions, including Washing, Checking, Doubting, Ordering, Obsessing, and Hoarding. Participants rate the distress caused by these symptoms on a 5-point scale, ranging from 0 (not at all) to 4 (extremely). The OCI provides both a total score and subscale scores, offering detailed insights into the severity and profile of OCD symptoms. The tool is noted for its clinical validity and reliability in both clinical and non-clinical samples.

To measure the severity of depressive symptoms specifically in women during the perinatal period, the Edinburgh Postnatal Depression Scale (EPDS) was administered [20]. This 10-item scale is specifically designed to identify women who have possible depression following childbirth. Each item is scored on a scale of 0 to 3, with higher total scores indicating more severe depressive symptoms. The EPDS is sensitive to changes over time and has been validated in numerous studies, making it an essential instrument for assessing PPD.

The State-Trait Anxiety Inventory for Adults (STAIY) was employed to differentiate between the temporary condition of “state anxiety” and the more general and long-standing quality of “trait anxiety” [21]. This tool includes separate self-report scales for assessing transient and enduring levels of anxiety. Each scale consists of 20 items rated on a 4-point scale, providing a quantitative measure of anxiety severity.

### 2.4. Statistical Analysis

Initial descriptive statistics (mean, standard deviation) and frequency distributions were computed for all demographic and clinical variables. Chi-square tests for categorical variables and t-tests for continuous variables were utilized to compare demographic characteristics between cases and controls, as well as antepartum versus postpartum conditions. Correlation coefficients (Pearson’s correlation coefficient) were calculated to explore the relationships between the continuous variables such as OCI scores, NEO-FFI subscales, and STAIY outcomes, providing insights into how these measures interconnect within the framework of PPD. Subsequently, significant predictors identified through correlation analyses were included in a logistic regression model with Edinburgh Depression Score as the dependent variable to quantify their impact on the risk and severity of PPD. The model adjusted for potential confounders identified during the univariate analyses. Statistical significance was set at an alpha level of 0.05. All analyses were conducted using SPSS Statistics software, version 25.0 (IBM Corp., Armonk, New York, NY, USA), ensuring robust data handling and accurate result computation.

## 3. Results

In this study, a comparative analysis between postpartum cases (*n* = 47) and controls (*n* = 49) was conducted with respect to various demographic and behavioral characteristics. The analysis revealed no significant difference in the mean age between cases and controls, with cases averaging 32 years (SD = 12.1) and controls 29 years (SD = 14.3), yielding a *p*-value of 0.271. However, significant differences were noted in the age distribution across the categories. Specifically, a higher proportion of younger women (20–24 years) were observed in the control group (28.57%) compared to the cases (6.38%), and a reverse trend was noted in the 30–34 years category, where cases (42.55%) significantly outnumbered controls (6.12%). This disparity across age groups was statistically significant (*p* < 0.001).

Significant variations were also observed in the background characteristics of the study participants. Urban living was more prevalent among controls (85.7%) than cases (63.8%), with a significant *p*-value of 0.013. Furthermore, educational attainment showed marked differences; the majority of controls held a university degree (97.95%) compared to cases (51.06%), and no cases had only a middle-school education compared to controls, which was statistically significant (*p* < 0.001). Substance use patterns between the groups also differed significantly. Alcohol consumption was higher in controls (28.57%) than in cases (10.64%), with a *p*-value of 0.027, and similarly, smoking was more prevalent among cases (17.0%) compared to controls (4.08%), with a *p*-value of 0.038 (Table 1).

Regarding anxiety levels measured by the STAIY, no significant differences were observed between the groups. The mean state anxiety scores were nearly identical for cases (36.51) and controls (35.26), resulting in a *p*-value of 0.551. Similarly, trait anxiety scores were comparable between cases (35.74) and controls (36.0), with a *p*-value of 0.897. However, significant differences were observed in several dimensions of obsessive-compulsive symptoms assessed by the OCI. Specifically, the scores for “Ordering” and “Hoarding” were notably higher among cases than controls, with *p*-values of 0.001 and 0.003, respectively. Cases had an average Ordering score of 5.89 (±4.94) compared to 3.44 (±3.18) in controls, and an average Hoarding score of 2.57 (±2.78) versus 1.16 (±1.65) in controls.

The total OCI score, representing the overall severity of obsessive-compulsive symptoms, also showed a significant difference, with cases averaging 29.1 (±23.5) compared to 19.0 (±17.8) in controls (*p* = 0.019), as presented in Table 2.

Neuroticism scores were significantly higher in cases (52.14 ± 27.46) compared to controls (38.87 ± 28.77), with a *p*-value of 0.023. Conversely, the subscale for Openness to Experience showed a notably different pattern, where cases had significantly lower scores (41.10 ± 24.60) compared to controls (62.51 ± 23.60), with a *p*-value of less than 0.001. No significant differences were found in the other three personality traits: Extraversion, Agreeableness, and Conscientiousness. The mean scores for Extraversion (cases: 49.72 ± 28.10; controls: 47.02 ± 31.97), Agreeableness (cases: 71.00 ± 28.13; controls: 69.06 ± 24.14), and Conscientiousness (cases: 63.04 ± 27.04; controls: 65.71 ± 30.67) indicated a relatively similar distribution across both groups (Table 3).

For the STAIY results, there was a statistically significant increase in state anxiety scores from the antepartum (36.51) to the postpartum period (41.55) with a *p*-value of 0.016. Similarly, the changes in trait anxiety increased significantly from 35.74 antepartum to 39.98 postpartum (*p*-value = 0.049). Regarding the OCI scores, no statistically significant changes were observed in the specific obsessive-compulsive symptoms from antepartum to postpartum in the domains for checking, doubting, obsessing, hoarding, and mental neutralizing, showing minimal variation. Notably, the washing score increased significantly from 7.53 to 8.91 (*p*-value = 0.006), and the ordering score increased from 5.64 to 6.79 (*p*-value = 0.008). The total OCI score increased from 27.1 to 32.9, yet these changes did not reach statistical significance (*p*-value = 0.248), as presented in Table 4.

Statistically significant increases were observed in the EPDS scores for anxiety and depression. Anxiety scores rose from a mean of 3.51 (±2.03) antepartum to 4.46 (±1.95) postpartum, with a *p*-value of 0.021. Similarly, depression scores increased from 8.11 (±4.29) to 9.93 (±4.20) with a *p*-value of 0.040. In contrast, the increase in suicidal thoughts, although rising from 0.06 (±0.31) to 0.17 (±0.44), did not reach statistical significance (*p*-value = 0.164). Analysis of the SVAA scores revealed no statistically significant changes postpartum in the domains of worrying about the newborn, worrying about partner satisfaction, and worrying about finances (Table 5). Worrying about self decreased significantly from 4.77 to 4.13 (*p*-value = 0.039).

**Figure 1:**
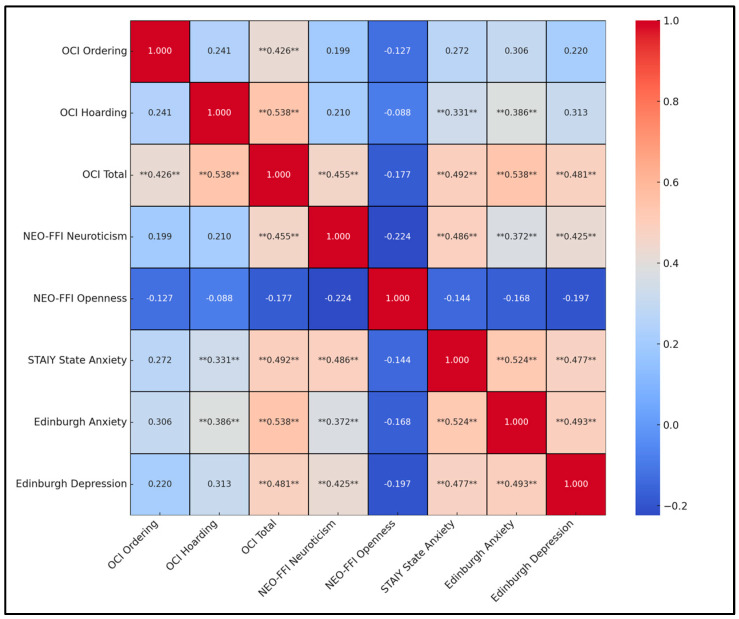
Correlation heatmap (**—Statistically significant correlations).

The total OCI score strongly correlated with specific OCD symptoms like ordering and hoarding (rho = 0.426 and rho = 0.538, respectively; both *p* < 0.001). Moreover, neuroticism, as measured by the NEO Five-Factor Inventory (NEO-FFI), was moderately and significantly correlated with the total OCI score (rho = 0.455, *p* = 0.004). In terms of psychological distress, both state anxiety and Edinburgh anxiety were significantly correlated with the total OCI score (rho = 0.492 and rho = 0.538, respectively; *p* < 0.006 and *p* < 0.003), reinforcing the connection between increased OCD symptomatology and elevated anxiety levels. The correlation matrix also revealed a significant link between neuroticism and both forms of anxiety as well as depression, with the highest correlation noted between neuroticism and state anxiety (rho = 0.486, *p* < 0.001). Additionally, Edinburgh depression scores were closely tied to the total OCI score (rho = 0.481, *p* = 0.007), as presented in Table 6 and Figure 1.

The analysis revealed a statistically significant relationship between the predictors and postpartum depressive symptoms. The intercept of the model was −3.15 with a standard error of 0.95, showing a significant effect (z = −3.32, *p* < 0.001) with a confidence interval ranging from −5.01 to −1.29. Neuroticism had a positive coefficient of 0.24 with a standard error of 0.07. This was statistically significant (z = 3.43, *p* < 0.001) and suggests that higher levels of neuroticism are associated with an increased likelihood of postpartum depression. The confidence interval for neuroticism ranged from 0.11 to 0.38. Conversely, openness had a negative coefficient of −0.13 with a standard error of 0.05, indicating that higher openness is associated with a lower likelihood of perinatal depression (z = −2.66, *p* = 0.009). The confidence interval for openness was between −0.23 and −0.03.

Edinburgh Anxiety showed a strong positive relationship with perinatal depression, with a coefficient of 0.51 and a standard error of 0.11. This relationship was highly significant (z = 4.64, *p* < 0.001), with a confidence interval from 0.29 to 0.73. Lastly, the total score on the OCI had a small but significant positive coefficient of 0.03 with a standard error of 0.01 (z = 2.97, *p* = 0.003), with a confidence interval ranging from 0.01 to 0.05 (Table 7).

## 4. Discussion

This study provides valuable insights into the predictors of PPD, highlighting the significant roles of personality traits and obsessive-compulsive symptoms. A critical finding of our analysis is the positive association between neuroticism and the severity of perinatal depression, which corroborates the existing literature suggesting that individuals with high neuroticism are more susceptible to depressive disorders due to their tendency to experience negative emotional states more frequently. The significant positive coefficient for neuroticism in our logistic regression analysis reinforces its predictive value for perinatal depression, underscoring the importance of screening for this personality trait in prenatal assessments.

Conversely, openness exhibited a protective effect against perinatal depression. The negative relationship between openness and PPD suggests that individuals who are more open to experience may possess better coping mechanisms and a more robust mental framework to manage the challenges of motherhood. This finding encourages further exploration into how the nurturing of openness traits before and during pregnancy might contribute to preventive strategies against PPD.

Additionally, the study identified a strong link between Edinburgh Anxiety scores and the likelihood of PPD, indicating that higher anxiety levels during the postpartum period are significant predictors of depressive symptoms. This relationship highlights the need for integrated treatment approaches that address both anxiety and depression as interconnected elements of postpartum mental health issues. The substantial correlations between OCD symptomatology—particularly ordering and hoarding—and PPD severity further suggest that OCD could exacerbate or trigger depressive symptoms in the postpartum period, warranting a comprehensive clinical approach that considers the full spectrum of obsessive-compulsive behaviors in maternal mental health evaluation.

Other studies demonstrate that certain personality traits significantly elevate the risk of perinatal depression. Puyané et al.’s meta-analysis [22] quantitatively establishes neuroticism as a strong predictor of PPD, with an odds ratio (OR) of 1.37 (95% CI: 1.22–1.53, *p* < 0.001), suggesting that higher levels of neuroticism correlate with a greater likelihood of developing PPD. Similarly, Verkerk et al.’s prospective study [23] not only confirms the influence of high neuroticism but also reveals that the combination of high neuroticism and introversion significantly predicts clinical depression across the first year postpartum, with increasing odds ratios at 3, 6, and 12 months postpartum (ORs: 3.08, 4.64, and 6.83, respectively). These objective findings underscore the need for targeted prenatal screening and interventions that consider these personality dimensions to prevent or mitigate the impact of PPD.

In the same manner, Iliadis et al. [24] provide compelling evidence that high levels of neuroticism during late pregnancy are robust predictors of PPDS, with adjusted odds ratios indicating a nearly fourfold increase in risk both at 6 weeks (aOR = 3.4, 95% CI: 1.8–6.5) and 6 months (aOR = 3.9, 95% CI: 1.9–7.9) postpartum. Additionally, other traits such as somatic and psychic trait anxiety also show significant associations with PPDS. Similarly, Murakami et al. [25] found that higher neuroticism scores are associated with PPDS and its dimensions (depressed mood, anxiety, anhedonia), while lower extraversion correlates with higher PPDS risks (OR = 0.74, 95% CI: 0.70–0.78). These findings suggest a consistent pattern across diverse populations where specific personality traits, especially neuroticism, significantly forecast the risk of developing PPDS. Both studies underscore the importance of assessing personality traits as part of prenatal care to better identify women at higher risk for PPDS, allowing for targeted interventions aimed at mitigating this risk.

The studies by Wakamatsu et al. [26] and Han et al. [27] offer insightful analyses into the predictors and mediators of perinatal depression (PPD). Wakamatsu et al. found that schizoid and melancholic temperaments, as measured by the TEMPS-A/MPT, alongside marital dissatisfaction from the PDPI-R, significantly predict PPD, with 10% of the study participants developing PPD. In contrast, Han et al. identified that maternal self-efficacy partially mediates the impact of vulnerable personality traits on PPD, explaining 29.0% of the variance in PPD outcomes, with self-efficacy alone accounting for an 18.0% mediation effect. Both studies highlight the importance of psychological and interpersonal factors in predicting and managing PPD, emphasizing the need for targeted interventions during pregnancy to mitigate risk.

Similarly, Terrone et al. [28] demonstrated that recent stressful life events and conscientiousness were significant predictors of depression, explaining 25.5% of the variance in pregnant women and 20.7% in postpartum women. Additionally, in pregnant women, openness, body dissatisfaction, and anxiety symptoms collectively accounted for more variance, with openness alone explaining 11.6%. In contrast, Roman et al. [29] focused on how personality traits influenced perinatal depression, mediated by perinatal anxiety. Their analysis revealed that neuroticism, when mediated by anxiety, significantly contributed to perinatal depression, with these relationships varying depending on the type of childbirth. Specifically, in the cesarean group, neuroticism and agreeableness were associated with higher depression scores, highlighting the nuanced interplay between personality, anxiety, and childbirth method in influencing perinatal mood disorders.

The study by Dudek et al. [14] explored the overlap between bipolar spectrum features and PPD and identified distinguishing personality traits in women with PPD symptoms. Utilizing standardized assessment tools like the Edinburgh Postnatal Depression Scale and Mood Disorder Questionnaire, the study revealed a significant correlation between PPD symptoms and bipolar features, particularly highlighting an increased neuroticism in those with ‘bipolar’ PPD. This suggests that neuroticism may serve as a potential marker for bipolarity in PPD, emphasizing the need for nuanced diagnostic approaches to effectively differentiate and treat ‘bipolar’ and ‘unipolar’ forms of PPD.

Several limitations are worth mentioning. The sample size, while adequate to reach statistical significance, was relatively small and geographically limited to one hospital setting in Romania, which may affect the generalizability of the findings to broader populations. The cross-sectional design also restricts the ability to infer causality between the observed psychological traits and perinatal depression. Another limitation that has to be acknowledged is the use of EPDS itself, as a tool that showed high variation in cutoff scores and positive predictive value across studies that evaluated it [30]. Additionally, the reliance on self-reported measures introduces the possibility of response bias, potentially influencing the accuracy of the reported severity of OCD symptoms and personality traits. Not evaluating the fathers involved within the families that participated in this study represents another limitation, as their influence on mothers’ mental health can be a confounding factor. Future research would benefit from larger, longitudinal studies that can track changes over time and utilize a multi-center approach to enhance the diversity and representativeness of the study sample.

## 5. Conclusions

The findings of this study demonstrate the intricate relationships between personality traits, OCD symptoms, and their impact on the severity of perinatal depression. By establishing significant predictors such as neuroticism, openness, anxiety, and OCD symptoms, this research contributes to the existing knowledge base and supports the development of targeted screening and intervention strategies. The identification of these key factors offers a pathway to enhance preventative measures and therapeutic approaches, potentially improving outcomes for women at risk of PPD.

## Figures and Tables

**Table 1:** Background characteristics of the cases and controls.

Variables	Cases (*n* = 47)	Controls (*n* = 49) *	*p*-Value
Age (mean ± SD)	32 ± 12.1	29 ± 14.3	0.271
Age range			<0.001
20–24 years	3 (6.38%)	14 (28.57%)	
25–29 years	10 (21.27%)	23 (46.93%)	
30–34 years	20 (42.55%)	3 (6.12%)	
>34 years	14 (29.78%)	9 (18.36%)	
Background			
Place of living (urban)	30 (63.8%)	42 (85.7%)	0.013
Work (employed)	42 (89.4%)	40 (81.6%)	0.283
Education			<0.001
Middle-school	3 (6.38%)	0 (0.00%)	
High-school	20 (42.55%)	1 (2.04%)	
University	24 (51.06%)	48 (97.95%)	
Substance use			
Alcohol	5 (10.64%)	14 (28.57%)	0.027
Smoking	8 (17.0%)	2 (4.08%)	0.038

*—Antepartum measurements; SD—Standard Deviation.

**Table 2:** OCI and STAIY survey results compared between cases and controls.

Variables (Mean ± SD)	Cases (*n* = 47)	Controls (*n* = 49) *	*p*-Value
STAIY			
State Anxiety	36.51 ± 9.63	35.26 ± 10.78	0.551
Trait Anxiety	35.74 ± 10.11	36.0 ± 9.58	0.897
OCI			
Washing	7.53 ± 2.37	6.12 ± 5.62	0.115
Checking	5.40 ± 5.82	3.48 ± 4.61	0.075
Doubting	1.85 ± 2.17	1.38 ± 1.67	0.236
Ordering	5.64 ± 2.11	3.44 ± 3.18	0.001
Obsessing	3.12 ± 3.65	2.46 ± 3.61	0.375
Hoarding	2.57 ± 2.78	1.16 ± 1.65	0.003
Mental Neutralizing	2.87 ± 3.26	1.87 ± 1.98	0.071
Total	27.1 ± 23.5	19.0 ± 17.8	0.019

*—Antepartum measurements; STAIY—State-Trait Anxiety Inventory for Adults, Form (higher scores indicate higher levels of anxiety); OCI—Obsessive-Compulsive Inventory (higher scores indicate greater severity of obsessive-compulsive symptoms).

**Table 3:** NEO-FFI survey results compared between cases and controls.

Subscales (Mean ± SD)	Cases (*n* = 47)	Controls (*n* = 49) *	*p*-Value
Neuroticism	52.14 ± 27.46	38.87 ± 28.77	0.023
Extraversion	49.72 ± 28.10	47.02 ± 31.97	0.661
Openness to Experience	41.10 ± 24.60	62.51 ± 23.60	<0.001
Agreeableness	71.00 ± 28.13	69.06 ± 24.14	0.717
Conscientiousness	63.04 ± 27.04	65.71 ± 30.67	0.652

*—Antepartum measurements; NEO-FFI—Five Factor Inventory.

**Table 4:** Comparison between antepartum and postpartum OCI and STAIY survey results.

Variables (Mean ± SD)	Antepartum (*n* = 47)	Postpartum (*n* = 47)	*p*-Value
STAIY			
State Anxiety	36.51 ± 9.63	41.55 ± 10.26	0.016
Trait Anxiety	35.74 ± 10.11	39.98 ± 10.54	0.049
OCI			
Washing	7.53 ± 2.37	8.91 ± 2.42	0.006
Checking	5.40 ± 5.82	5.40 ± 6.07	0.998
Doubting	1.85 ± 2.17	1.97 ± 2.43	0.799
Ordering	5.64 ± 2.11	6.79 ± 2.06	0.008
Obsessing	3.12 ± 3.65	2.97 ± 3.49	0.837
Hoarding	2.57 ± 2.78	2.17 ± 2.12	0.428
Mental Neutralizing	2.87 ± 3.26	2.78 ± 3.45	0.895
Total	27.1 ± 23.5	32.9 ± 24.9	0.248

STAIY—State-Trait Anxiety Inventory for Adults, Form (higher scores indicate higher levels of anxiety); OCI—Obsessive-Compulsive Inventory (higher scores indicate greater severity of obsessive-compulsive symptoms).

**Table 5:** Comparison between antepartum and postpartum Edinburg and SVAA scales results.

Variables (Mean ± SD)	Antepartum (*n* = 47)	Postpartum (*n* = 47)	*p*-Value
Edinburgh			
Anxiety	3.51 ± 2.03	4.46 ± 1.95	0.021
Depression	8.11 ± 4.29	9.93 ± 4.20	0.040
Suicidal thoughts	0.06 ± 0.31	0.17 ± 0.44	0.164
SVAA			
Worrying about self	4.77 ± 1.84	4.13 ± 1.01	0.039
Worrying about the newborn	6.08 ± 3.20	6.68 ± 3.34	0.371
Worrying about partner satisfaction	8.04 ± 2.68	8.12 ± 2.52	0.880
Worrying about finances	7.55 ± 2.54	7.48 ± 2.64	0.894

SVAA—Situational Vulnerability and Adjustment Assessment (higher scores indicate more significant worries or difficulties adjusting to new parenting roles and responsibilities); higher scores on the EPDS indicate greater severity of depressive symptoms.

**Table 6:** Correlation matrix.

Rho/*p*-Value	OCI Ordering	OCI Hoarding	OCI Total	NEO-FFI Neuroticism	NEO-FFI Openness	STAIY State Anxiety	Edinburgh Anxiety	Edinburgh Depression
OCI Ordering	1							
OCI Hoarding	0.241 (*p* = 0.112)	1						
OCI Total	0.426 (*p* < 0.001)	0.538 (*p* < 0.001)	1					
NEO-FFI Neuroticism	0.199 (*p* = 0.178)	0.210 (*p* = 0.143)	0.455 (*p* = 0.004)	1				
NEO-FFI Openness	−0.127 (*p* = 0.256)	−0.088 (*p* = 0.432)	−0.177 (*p* = 0.191)	−0.224 (*p* = 0.123)	1			
STAIY State Anxiety	0.272 (*p* = 0.083)	0.331 (*p* = 0.039)	0.492 (*p* = 0.006)	0.486 (*p* < 0.001)	−0.144 (*p* = 0.218)	1		
Edinburgh Anxiety	0.306 (*p* = 0.060)	0.386 (*p* = 0.021)	0.538 (*p* = 0.003)	0.372 (*p* = 0.009)	−0.168 (*p* = 0.195)	0.524 (*p* < 0.001)	1	
Edinburgh Depression	0.220 (*p* = 0.133)	0.313 (*p* = 0.053)	0.481 (*p* = 0.007)	0.425 (*p* < 0.001)	−0.197 (*p* = 0.171)	0.477 (*p* < 0.001)	0.493 (*p* < 0.001)	1

NEO-FFI—Five Factor Inventory; OCI—Obsessive-Compulsive Inventory; STAIY—State-Trait Anxiety Inventory for Adults, Form.

**Table 7:** Logistic regression results for depression and depressive symptoms postpartum.

Coefficient	Estimate	SE	z-Value	*p*-Value	95% CI Lower	95% CI Upper
**Intercept**	−3.15	0.95	−3.32	<0.001	−5.01	−1.29
**NEO-FFI Neuroticism**	0.24	0.07	3.43	<0.001	0.11	0.38
**NEO-FFI Openness**	−0.13	0.05	−2.66	0.009	−0.23	−0.03
**Edinburgh Anxiety**	0.51	0.11	4.64	<0.001	0.29	0.73
**OCI Total**	0.03	0.01	2.97	0.003	0.01	0.05

NEO-FFI—Five Factor Inventory; SE—Standard error; CI—Confidence Interval; OCI—Obsessive-Compulsive Inventory.

## Data Availability

Data available on request from the authors.
